# Management of prosthetic joint infections in France: a national audit to identify key situations requiring innovation and homogenization

**DOI:** 10.1186/s12879-021-06075-x

**Published:** 2021-05-01

**Authors:** Marion Le Maréchal, Zoé Cavalli, Cécile Batailler, Jean-François Gonzalez, André Ferreira, Sébastien Lustig, Tristan Ferry, Johan Courjon

**Affiliations:** 1 Centre Hospitalier de Grenoble, Grenoble, France; 2 Université de Grenoble-Alpes, Grenoble, France; 3 Hôpital de Mercy, Centre Hospitalier Régional de Metz-Thionville, Ars-Laquenexy, France; 4 Claude Bernard University Lyon 1, Villeurbanne, France; 5 Université Côte d’Azur, Nice, France; 6 Société Française de chirurgie de la hanche et du genou, Paris, France; 7 CRIOAc Lyon, Lyon, France; 8 Université Côte d’Azur, CHU, INSERM, C3M, Nice, France; 9 Infectiologie, Hôpital Archet 1, Centre Hospitalier Universitaire de Nice, Nice, France

**Keywords:** Arthritis infection, Clinical audit, Joint prosthesis, Rifampin, *Staphylococcus aureus*, Surveys and questionnaires

## Abstract

**Background:**

Prosthetic joint infections (PJI) are one of the most serious complication of arthroplasty. The management of PJI needs a multidisciplinary collaboration between orthopaedic surgeon, infectious disease specialist and microbiologist. In France, the management of PJI is organized around reference centres (CRIOACs). Our main objective was to perform an audit through a questionnaire survey based on clinical cases, to evaluate how French physicians manage PJI. Eligible participants were all physicians involved in care of patients presenting a PJI. Physicians could answer individually, or collectively during a multidisciplinary team meeting dedicated to PJI. The survey consisted as three questionnaires organized in a total of six clinical cases.

**Results:**

Answers from the CRIOACs to the three questionnaires were 92, 77, and 53%. Between 32 and 39% of respondents did not administer antibiotic prophylaxis despite positive *S. aureus* pre-operative documentation. One-stage exchange strategy was widely preferred in all clinical cases, with no difference between CRIOACs and other centres. Rifampicin was prescribed for *S. aureus* PJI, in a situation with (90–92%) or without any prosthesis (70%). There was no consensus for the total antibiotic regimen duration, with prescriptions from six to 12 weeks for a majority of respondents.

**Conclusions:**

Surgical strategy for the management of PJI was homogenous with a preference for a one-stage exchange strategy. Medical management was more heterogenous, which reflects the heterogeneity of those infections and difficulties to perform studies with strong conclusions.

**Supplementary Information:**

The online version contains supplementary material available at 10.1186/s12879-021-06075-x.

## Introduction

Prosthetic joint infections (PJI) are one of the most serious complication of arthroplasty [1]. Those infections are a complication following surgery, with a frequency of 0.8–1.9% of PJI after total knee replacement [2–4], and 0.3–1.7% of PJI after total hip replacement [4–6]. The management of PJI needs a multidisciplinary collaboration between, at least, orthopaedic surgeon, infectious disease specialist and microbiologist, to decide for exams to perform in the pre-operative evaluation, the type of surgery to choose, and the most suitable antibiotic regimen. Even if the benefit of such collaboration has not been published in PJI management, it is known in other complex diseases, such as endocarditis [7].

One of the main issues in PJI management is the high heterogeneity of clinical situation: heterogeneity on the localization, on the delay (early (1–3 months after implantation [1]), or delayed), and on the presentation (acute or chronic). Microbiologic samples can isolate, none, one, or several bacteria.

In the field of PJI recommendations, the level of evidence remains low. For instance, in the recommendations from IDSA concerning the management of PJI, 63.5% of recommendations are based on level III quality of evidence (evidence from opinions of respected authorities, based on clinical experience, descriptive studies, or reports of expert committees) [8].

In France, in 2008, hospitalization for PJI accounted for 0.2% of all hospitalization [9]. The management of PJI is mainly performed in public hospitals (83% in 2008, [9]), and organized around reference centres. Considering the heterogeneity of PJI to manage and the global low level of evidence available, we hypothesized that clinical practice was diversified from one hospital to another.

Our main objective was to perform an audit through a questionnaire survey based on clinical cases, to evaluate how French physicians declare managing PJI in daily care.

## Methods

### Background information

For PJI management France is organized, since 2008, in a large network of Reference centres specialized in the management of PJI *(Centre de Référence des Infections Ostéo-Articulaires complexes (CRIOACs)).* This CRIOACs network consists of nine reference centres, each coordinating at least two of the 21 associated centres.

All physicians and hospitals in France can contact one of the CRIOACs, to ask for a patient management advice, or for a patient transfer to a specialized centre. The list of all CRIOACs is available on the ministry of health website [10].

### Participants

Eligible participants were all physicians involved in care of patients presenting a PJI, with no restriction regarding their speciality (surgeons, clinical care medicine or microbiologists). We restricted the list to the physicians who subscribed to one of those emailing lists: 1) French society of hip and knee *(Société Française de la Hanche et du Genou (SFHG))*, 2) National network of research in infectious diseases *(Réseau National de Recherche Clinique en Infectiologie (RENARCI)),* 3) The CRIOAC centers emailing list. All physicians who subscribed to one (or several) emailing list(s) received an invitation email (sent by JC and TF) including information about the study and a link to the online survey. Total number of physicians who received the invitation email is too difficult to establish. Therefore, we estimated the response rate over the number of CRIOACs who answered among the total number of CRIOACs in France (*N* = 30).

Physicians could answer individually, or collectively during a multidisciplinary team meeting dedicated to PJI (one answer per meeting independently of the number of physicians taking part to the meeting).

### Survey tool development

**Fig. 1:**
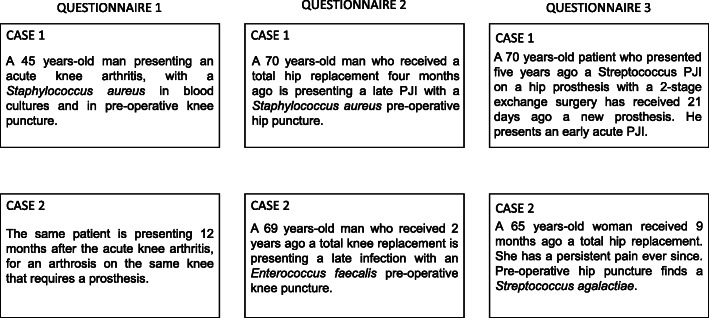
Summary of the six clinical cases. PJI: Prosthetic joint infection

The survey was divided in three questionnaires sent in a 5 months period. Each questionnaire was divided in three parts: 1) an introduction to characterize the participant(s); 2) the first clinical case; 3) the second clinical case (Fig. 1). Questionnaires are available in “Annexes” part). Those questionnaires were developed by JC, MLM, TF, and ZC based on the literature, on their personal experience, and on issues addressed during multidisciplinary meetings dedicated to PJI. Clinical situations were chosen for their frequency in clinical practice [11] for the lack of recommendations and literature in the situation, and for their diversity. We aimed to address common issues with and without prosthesis, late and early, and with different bacteria. However, we decided to limit algorithms in respondents’ choices to make the results easier to understand. Therefore, we did not include any situation involving reconstructive or vascular surgery.

Each questionnaire, was written in French, formatted in SurveyMonkey® and the first one was pilot tested by four physicians, to check for length and clarity.

Non-infectious diseases specialists could skip questions dedicated to antibiotic therapy with a dedicated answer “Not concerned with medical treatment decisions”.

### Data collection

The first questionnaire was opened on January 2019 (invitation sent the 14th of January 2019), the second one on February 2019 (invitation sent the 5th of March 2019), and the third one on May 2019 (invitation sent the 20th of May 2019). The three questionnaires were closed on the 30th of June 2019. Reminders for the previous questionnaires were sent in each email invitation. Participation in the survey was voluntary, anonymous and without any financial compensation.

### Ethical statement

All methods were carried out in accordance with the French Comission Nationale de l’informatique et des Libertés (CNIL) guidelines. All protocols were approved by the scientific committee of the French bone and joint infections national reference center network (CRIOAc). Informed consent was obtained from all health-care providers who participate to this survey.

### Data analyses

Data from thrice surveys were extracted from the SurveyMonkey® platform, imported in an Excel® spread sheet and analysed with the R® software (version 3.5.1). Continuous variables were presented as means (and standard deviations), or as medians (and interquartile ranges) depending on the variable’s distribution. Categorical variables were described as absolute numbers and percentages.

Univariate analyses were performed, using fisher exact test or chi square test were needed.

All answers were considered. If several physicians from the same institution answered individually (and not as a multidisciplinary team meeting), their answers were weighted to overcome the weight of one reference center compared to the weight of selection bias. For instance, when two physicians from the same institution answered individually, each of their answer weighted for 0.5).

## Results

All answers presented in the *Results* section are weighted data (details in *Data analyses* section). All *p*-values presented in the Results section are the result of univariate analysis.

### Baseline characteristics of respondents

We sent thrice questionnaires to the three emailing lists detailed in the *Participants* section. Answers from the CRIOACs to the questionnaires were: 1) 27.5/30 to the first questionnaire (Q1) (response rate = 92%); 2) 23/30 to the second questionnaire (Q2) (response rate = 77%); 3) 16/30 to the third questionnaire (Q3) (response rate = 53%). Respondents finished the questionnaires in 68% (Q1), 71% (Q2), and 67% (Q3) of cases. All answers were taken into consideration. All respondents’ characteristics are available Table 1.

**Table 1:** Characteristics of respondents (weighted data)

	First questionnaire		Second questionnaire		Third questionnaire	
	n/N	%	n/N	%	n/N	%
**The hospital is a CRIOAC in the management of PJI?**						
*Yes*	27.5/63	43.7	23/50	46.0	16/56	28.6
*No*	34.5/63	54.8	25/50	50.0	38/56	67.9
**If the hospital is one of the CRIOAC in the management of PJI, how many multidisciplinary meetings dedicated to PJI are organized (per month)?**						
*< 4*	5.5/27.5	20.0	7.5/23	32.6	1.5/16	9.4
*4*	17.75/27.5	64.5	10.5/23	45.7	8/16	50.0
*> 4*	5/27.5	18.2	4/23	17.4	4.5/16	28.1
**If the hospital is not one of the CRIOAC in the management of PJI, do you have a multidisciplinary meeting dedicated to PJI?**						
*Yes*	10.5/34.5	30.4	8.5/25	34.0	17.5/38	46.1
*No*	22/34.5	63.8	15.5/25	62.0	20.5/38	53.9
**For the hospital that are not one of the CRIOAC in the management of PJI, how many multidisciplinary meetings dedicated to PJI are organized (per month)?**						
*< 4*	5.5/10.5	52.4	5/8.5	58.8	10.5/17.5	60.0
*4*	2/10.5	19.0	2/8.5	23.5	5/17.5	28.6
*Other*	1/10.5	9.5	1.5/8.5	17.6	1/17.5	5.7
**For the hospital that are not one of the CRIOAC in the management of PJI, do you ask for expertise to a CRIOAC?**						
*Yes*	22.5/34.5	65.2	16/25	64.0	27.5/38	72.4
*No*	7/34.5	20.3	8/25	32.0	10.5/38	27.6
**Do you answer as an individual person or during a multidisciplinary meeting dedicated to PJI?**						
*Individual answer*	41/63	65.1	29/50	58.0	48/56	85.7
*Multidisciplinary meeting*	16/63	25.4	15.5/50	31.0	6/56	10.7
**For individual respondents, what is your speciality?**						
*Surgeon*	32/41	78.0	25.5/29	87.9	37/48	77.1
*Infectious disease specialist*	5.5/41	13.4	4.5/29	15.5	10/48	20.8
*Other*	3.5/41	8.5	1/29	3.4	1/48	2.1
**If you are answering during a multidisciplinary team meeting, who are the specialist participating to the meeting?**						
*Only surgeon, infectious disease specialist, and microbiologist*	7/16	43.8	5.5/15.5	35.5	1.5/6	25.0
*More than surgeon, infectious disease specialist and microbiologist*	7/16	43.8	9/15.5	58.1	1/6	16.6
*Other*	2/16	12.5	1/15.5	6.5	3.5/6	58.3
**How many PJI do you manage?**						
*Less than one a month*	24/63	38.1	20/50	40.0	8.5/56	15.2
*1 to 5 a month*	8/63	12.7	4/50	8.0	12/56	21.4
*1 to 5 a week*	7.75/63	12.3	6.5/50	13.0	5.5/56	9.8
*More than 5 a week*	16.75/63	26.6	15/50	30.0	26.5/56	47.3

CRIOAC: Reference centre in the management of PJI; *PJI* Prosthetic joint infection

### Prophylaxis of PJI

In a situation with a patient presenting an history of invasive *S. aureus* infection (acute arthritis of a native joint associated with an MSSA bacteraemia, Q1C1 and Q1C2), 94% of respondents (50.75/54.25) were not performing a systematic nasal decolonization with mupirocin (without performing anterior nare swab), and 42% (22.75/54.25) were detecting *S. aureus* from anterior nare swab and treating the carrying patients.

For a patient with a history of *S. aureus* acute arthritis who requires a prosthesis on the same knee (Q1C2), 32% (17.5/54.25) did not administer antibiotic prophylaxis, with no difference between CRIOACs (25%, 6.5/25.5) and other centres (36%, 10/27.75), *p*-value = 0.406. For a patient presenting a late PJI with a positive *S. aureus* pre-operative needle puncture, in case of 1-stage exchange strategy, 39% (13.5/34.5) did not administer antibiotic prophylaxis, with a significant difference between CRIOACs (26%, 4.5/17.5), and other centres (67%, 9/13.5), *p*-value = 0.023.

### Surgical intervention

Concerning late PJI (Q2C1, Q2C2, and Q3C1), most of respondents chose a 1-stage exchange strategy (72–91%) compared to 2-stage exchange strategy (9–20%) or debridement with removal of mobile components via an open arthrotomy (23%) (Table 2).

**Table 2:** Surgical management of prosthetic and joint infections (weighted data)

		Q2C1	Q2C2	Q3C1	Q3C2
**STRATEGY**	DAIR	2% (1/44.5)	5% (2/40)	23% (11/47)	21% (11/53.5)
	1-stage	91% (40.5/44.5)	75% (30/40)	82% (38.5/47)	72% (38.5/53.5)
	2-stage	4% (2/44.5)	20% (7/40)	9% (4/47)	7% (4/53.5)
**CEMENT**^a^	1-stage		88% (26.5/30)		42% (16.5/38.5)
	2-stage		47% (7/15)		8% (3/38,5)

^a^Use of antibiotic-loaded cement

Concerning early PJI (Q3C1), 94% of respondents (48.5/51.5) chose a debridement with removal of mobile components via an open arthrotomy, 4% (2/51.5) chose a 1-stage exchange strategy, 2% (1/51.5) chose a debridement via an arthroscopy without any removal, and none of the respondents chose a 2-stage exchange strategy.

There was no difference between CRIOACs and other centres to choose between a 1-stage or a 2-stage exchange strategy (Q2C2: 22% (4/18.5) of 2-stage exchange strategy for non CRIOACs vs. 21% (4/19.5) for CRIOACs, *p*-value = 1; Q3C2: 5% (1.5/27.5) vs. 22% (2.5/11.5), *p*-value = 0.562). The 1-stage exchange strategy was more chosen for the *S. aureus* infections. There was a significant difference between the choice of a 1-stage exchange strategy for a *S. aureus* PJI and a *E. faecalis* PJI (*p* = 0.02), and between a *S. aureus* and a gram-positive cocci in chains documentation (*p* = 0.007). However, there was no difference between CRIOAC and non-CRIOAC centres.

Antibiotic impregnated bone cement was used in case of 1-stage or 2-stage exchange strategy (Table 2).

### Post-operative antibiotic treatment

Concerning MSSA bone and joint infection (BJI)/PJI (Q2C1, and Q3C1) for a late chronic PJI, after a positive *S. aureus* pre-operative needle puncture, 22% (10/44.5) of respondents did not consider this positive puncture to choose post-operative antibiotic therapy.

Concerning late PJI with a pre-operative documentation (Q2C2 and Q3C2), most respondents chose a broad-spectrum antibiotic (Table 3), and 60% (18.5/31) used a dual antibiotic regimen.

**Table 3:** Medical treatment for prosthetic and joint infections

		Q1C1	Q1C2	Q2C1	Q2C2	Q3C1	Q3C2
Post-operative treatment	Pre-operative documentation	YES	NO	YES	YES	NO	YES
	Bacteriological documentation	MSSA	MSSA	SAMS	*E. faecalis*	MSSA	*S. agalactiae*
	Narrow-spectrum antibiotic	99% (36.25/36.5)	NA	NA	24% (12/50)	NA	45% (14/31)
	Broad-spectrum antibiotic	0%	NA	NA	43% (21.5/50)	NA	52% (16/31)
Oral antibiotic	Rifampicine + quinolones	70% (28/40)	92% (33.5/36.5)	NA	NA	90% (19/21)	NA
Total antibiotic regimen after DAIR	3 weeks	6% (2/36)	NA	NA	NA	NA	
	4 weeks	35% (12.5/36)					
	6 weeks	53% (0.5/36)					26% (6/23.5)
	8 weeks	1% (0.5/36					
	3 months	3% (1/36)					68% (16/23.5)
	Suspensive	NA					4% (1/23.5)

*DAIR* Debridement, Antibiotic and Implant Retention, *MSSA* Methicillin-susceptible Staphylococcus aureus, *NA* Not applicable

### Oral antibiotic regimen

Concerning the prescription of rifampicin for *S. aureus* BJI/PJI (Q1C1, Q2C1, and Q3C1): respondents were giving priority to a regimen associating fluoroquinolone and rifampicin (70–90%) (Table 3).

For *S. aureus* PJI (Q2C1), clindamycin was a frequent choice if fluoroquinolone or rifampicin were not available (64%, 23.5/36.5), but the MLS_B_ inducible phenotype was considered (23%, 8.5/36.5 of clindamycin in case of a MLS_B_ inducible phenotype), then cotrimoxazole (7%, 2.5/36.5) was the first alternative.

For *S. agalactiae* PJI the use of dual therapy was common (91%, 36/39.5). Dual therapy including rifampicin was chosen among 44% (18.5/39.5) of the responders.

### Total antibiotic regimen duration

Concerning total antibiotic regimen duration: 1) After a 1-stage exchange surgery, total antibiotic regimen duration was for Q2C1: 4–6 weeks (16%, 6/36.5), 6–8 weeks (49%, 18/36.5), 8–12 weeks (33%, 12/36.5), or 6 months (1%, 0.5/36.5); and for Q3C2: 6 weeks (37%, 16.5/45), 8 weeks (4%, 2/45), or 12 weeks (59%, 26.5/45).

2) After a 2-stage exchange surgery (Q2C2), total antibiotic regimen duration was 8 weeks (6 weeks between explantation and implantation, and 2 weeks after implantation) (45%, 19/42), 10 weeks (5%, 2/42), or 12 weeks (6 weeks between explantation and implantation, and 6 weeks after implantation) (24%, 10/42).

3) After a debridement via an arthroscopy without mobile components exchange (Q1C1 and Q3C1), total antibiotic regimen duration varied between 3 weeks and 3 months (Table 3).

## Discussion

### Surgical management

The 2-stage exchange strategy is commonly used in the USA and is considered as the gold standard for prosthesis replacement [1, 12]. An European survey (EBJIS survey) also found that 2-stage exchange strategy was the most common philosophy regarding treatment of chronic PJI [13]. However, when prosthesis replacement was clearly indicated for chronic infections in our study, 1-stage exchange strategy was chosen more frequently (without any significant difference between CRIOACs and other centres). Our clinical cases presented PJI without sinus tract or inadequate soft tissue coverage, no systemic manifestation of infection and with identified organisms prior to surgery; none of the usual criteria for a 2-stage exchange strategy were present [14]. More recently, a better functional outcome of 1-stage exchange strategy has been suggested in total knee arthroplasty [15, 16]. Those results need to be confirmed but they highlight another crucial outcome criteria which has to be considered beside the microbiological cure.

### IV antibiotic therapy

In case of a positive *S. aureus* pre-operative needle puncture (Q2C1), 22% of respondents did not consider this positive result to choose the post-operative antibiotic therapy. For a late *S. agalactiae* PJI, 12% started an antibiotic therapy before surgery, based on the pre-operative needle puncture. Even with the pre-operative identification of a bacteria (*E. faecalis* in Q2C2, or *S. agalactiae* in Q3C2), 45 and 47% of respondents, added an antibiotic therapy against MRSA to the penicillin therapy. Few studies have compared the microbiologic concordance between pre-operative and per-operative samples. In the work of Goulenok et al., only presented in congress, concordance between both exams was 85% [17], and in a Matter-Parrat et al. work, concordance was 74% [18]. The choice, whether to start or not an antibiotic regimen before surgery, and therefore before per-operative microbiological samples probably needs to be decided depending on the isolated bacteria after needle puncture (commensal or strictly pathogenic bacteria) and, as suggested by the Spanish recommendations, in case of a skin and soft tissue infection, an antibiotic therapy can be started for few days, in order to allow a non inflammatory surgical approach [19].

In two clinical cases (Q1C2 and Q2C1), questions on antibiotic prophylaxis showed that respectively 32 and 39% of respondents did not prescribe antibiotic prophylaxis before performing microbiological sample. The main reason is probably the risk to sterilise microbiological samples. A literature review from Wouthuyzen-Bakker et al. pooled all studies comparing patient with and without antibiotic prophylaxis [20]. In the antibiotic prophylaxis group, 88% of cultures were positive, vs. 95% in the non antibiotic prophylaxis group (*p*-value = 0.004). However, several works showed that after a PJI treatment, a failure can occur with the same bacteria, or with another one, which enhance the importance of all infections prevention. Giving the functional damages and the financial burden of PJI, from our point of view it seems more prudent to administer antibiotic prophylaxis at the cost of microbiological samples.

### Oral antibiotic prescription

Rifampicin was preferred in all situation with a *S. aureus* PJI (Q2C1 and Q3C1), with respectively 92 and 90% of respondents who were giving priority to an oral regimen associating fluoroquinolone and rifampicin. The crucial role of rifampicin for PJI treatment has already been demonstrated several times [21]. Recommendations on this regimen for *S. aureus* are clear with a high level of evidence (A-I) [1, 19]. The presence of an MLS_B_ inducible phenotype in *S. aureus* significantly influence the selection of antibiotics in our survey. However, data regarding clindamycin resistance emergence in such situation in still lacking and a recent report from a French team analysing the outcome of *S. aureus* BJI with such phenotype treated with clindamycin is reassuring when another antibiotic is associated [22]. Finally, for *S. aureus,* cotrimoxazole, clindamycin and fusidic acid are the three drugs chosen after rifampin and fluoroquinolone. This is in accordance with the body evidence of antibiotic diffusion in bone and the accumulation of efficacy data of those drugs for BJI [23]. Despite appropriate bone diffusion linezolid is the last choice probably because of its safety profile and the fear of adverse effects promoted by an off-label use beyond 4 weeks. Nevertheless, because of an increased tolerability [24] it will be interesting to follow the place of tedizolid in this context.

Despite a significant impact on the outcome [25] in the largest cohort published to date rifampicin was not included in the majority of treatment proposition for of *S. agalactiae* PJI.

### Total duration of antibiotic regimen

In our survey, after a 1-stage exchange surgery, total antibiotic regimen duration was from 6 to 8 weeks (49%, Q2C1), or 12 weeks (59%, Q3C2). After a 2-stage exchange surgery, total antibiotic regimen duration was 8 weeks (including 2 weeks after reimplantation) (45%, Q2C2). After a debridement via an arthroscopy without mobile components exchange (Q3C1) 3 months (68%). A recent literature review by Yen et al., in 2019 analysed 10 studies that compared short-course (4 to 8 weeks) versus long-course (4 weeks to 6 months) antibiotic regimen [26]. It included 856 patients from 1987 to 2013. They did not find any difference between both groups (RR = 0.87, 95% confidence interval (CI) [0.62–1.22]). For *S. aureus* PJI, after a 1-stage exchange surgery, American recommendations suggest a total duration of antibiotic regimen of 3 months [1]; Spanish recommendations are more moderate (and more recent), and suggest a total antibiotic regimen going from four to 8 weeks [19], based on a literature review over 28 studies, but with a low level of evidence (B-II). A French work, DATIPO, published its first results [27]. They compared 6 weeks vs. 12 weeks antibiotic therapy for PJI. They did not show non-inferiority in the 6 weeks group, with 22% vs. 14% of failure (8.2 90%CI [0.7–15.7]). Based on these results, 3 month of therapy seems to be more appropriate.

#### Strengths and limitations

Our study is a pilot study. It is original, since it was dedicated to all physicians involved in the management of PJI, and because we distinguished CRIOACs in the management of PJI with other centres, and individual answers with answers during a multidisciplinary meeting dedicated to PJI. The survey was sent through three different emailing lists and therefore touched the utmost concerned physicians. The data were weighted to overcome the selection bias and to avoid large centres to be over-represented.

Our work also has some limitations. First, the response rate is hard to evaluate since we do not know precisely how many physicians received the invitation emails. The response rate from the CRIOACs decreased during time (92, 77 and 53%). This is probably due weariness, since our survey was spread over few months. Then, the characteristics of the respondents to the third questionnaire were very different to those in the first and second questionnaires. More respondents were answering as individuals, and less respondents were part of CRIOACs.

## Conclusion

Surgical strategy for the management of PJI with common clinical and bacteriological backgrounds was homogenous with a preference for one-stage strategy. Medical management was more heterogenous with a wide range of duration of post-operative antibiotic duration, or total antibiotic duration. This heterogeneity in medical PJI management reflects the heterogeneity of those infections and difficulties to perform studies with strong conclusions, and therefore to publish high level of evidence recommendations.

## Supplementary Information

**Additional file 1.**

**Additional file 2.**

**Additional file 3.**

## Data Availability

The Datasets used and analysed during the current study available from the corresponding author on reasonable request.
