# Supplementary material for: Management of prosthetic joint infections in France: a national audit to identify key situations requiring innovation and homogenization
Source: BMC Infect Dis. 2021 May 1;21:401. doi: 10.1186/s12879-021-06075-x (PMC8088082; doi:10.1186/s12879-021-06075-x)
Supplement: Supplementary file 1 — Additional file 1. [file 12879_2021_6075_MOESM1_ESM.pdf]

Title: Management of prosthetic joint infections in France: a national audit to identify key situations requiring innovation and homogenization

Supplementary material questionnaires

Authors

Marion Le Maréchal[1-2], Zoé Cavalli[3], Cécile Batailler[4], Jean-François Gonzalez[5], André Ferreira[6], Sébastien Lustig[4], Tristan Ferry[4-7], Johan Courjon[8-9†] on the behalf of the scientific committee of CRIOAc

1. Service de Maladies Infectieuses, CHU de Grenoble
2. Université de Grenoble-Alpes
3. Service de Maladies Infectieuses, CHR de Metz-Thionville
4. CRIOAc Lyon
5. Service de chirurgie orthopédique et chirurgie du sport, Institut universitaire locomoteur et du sport (IULS), Hôpital Pasteur 2, Centre Hospitalier Universitaire de Nice, Nice, France
6. Société Française de chirurgie de la hanche et du genou
7. Université Claude Bernard Lyon 1, Lyon, France.
8. Infectiologie, Hôpital Archet 1, Centre Hospitalier Universitaire de Nice
9. Université Côte d'Azur, CHU, INSERM, C3M, France

[†]**Corresponding author:** Johan Courjon, email: courjon.j@chu-nice.fr

- Postal address: CHU de Nice, Hôpital Archet 1 Infectiologie 151 route de St Antoine de Ginestière 06200 NICE
- Phone number: +33 (0)4 92 03 54 61 Fax number +33 (0)4 92 03 90 66

Supplementary material 1 – Questionnaire 1

1. What is the name of the hospital you are working in?
   [Open answer]

2. Is your hospital a reference center in the management of prosthetic joint infection (PJI) *(Centre de Référence des Infections Ostéo-Articulaires complexes (CRIOACs))*
   - Yes
   - No

3. What is the name of your hospital?
   [Drop-down menu]

4. How many meetings dedicated to PJI are organized per month in your hospital?
   [Open answer]

5. Is there a meeting dedicated to PJI in your hospital?
   - Yes
   - No

6. How many meetings dedicated to PJI are organized per month in your hospital?
   [Open answer]

7. Do you refer to the reference center for an opinion on complex PJI?
   - Yes
   - No

8. What is the reference center you are referring to?
   [Drop-down menu]

9. Are you answering as an individual or during a multidisciplinary meeting dedicated to PJI?
   - Individual
   - Multidisciplinary meeting dedicated to PJI

10. What is your specialty?
    - Surgeon
    - Anesthetist
    - Infectious diseases specialist
    - Internal medicine specialist
    - Physical therapy physician
    - Rheumatologist
    - Microbiologist
    - Other [Open answer]

11. If you are a surgeon, what is your specialty?
    [Open answer]

Supplementary material 1 – Questionnaire 1

1. What is the name of the hospital you are working in?
   [Open answer]

2. Is your hospital a reference center in the management of prosthetic joint infection (PJI) *(Centre de Référence des Infections Ostéo-Articulaires complexes (CRIOACs))*
   - Yes
   - No

3. What is the name of your hospital?
   [Drop-down menu]

4. How many meetings dedicated to PJI are organized per month in your hospital?
   [Open answer]

5. Is there a meeting dedicated to PJI in your hospital?
   - Yes
   - No

6. How many meetings dedicated to PJI are organized per month in your hospital?
   [Open answer]

7. Do you refer to the reference center for an opinion on complex PJI?
   - Yes
   - No

8. What is the reference center you are referring to?
   [Drop-down menu]

9. Are you answering as an individual or during a multidisciplinary meeting dedicated to PJI?
   - Individual
   - Multidisciplinary meeting dedicated to PJI

10. What is your specialty?
    - Surgeon
    - Anesthetist
    - Infectious diseases specialist
    - Internal medicine specialist
    - Physical therapy physician
    - Rheumatologist
    - Microbiologist
    - Other [Open answer]

11. If you are a surgeon, what is your specialty?
    [Open answer]

12. If you are answering during a multidisciplinary meeting dedicated to PJI, what are the specialties of the attending physicians?
    [Open answer]

13. How many PJI do you manage?
    - More than 5 a week
    - 1 to 5 a week
    - 1 to 5 a month
    - Less than 1 a month

CLINICAL CASE N°1 (Q1C1)

You are called for an opinion on the management of Mr X, 45 years-old, without any comorbidities, who was admitted to the emergency department 24 hours ago for a 48 hours fever, a complete functional disability of the left knee (no prosthesis). During clinical examination, the knee is swollen, warm, painful, with a patellar tap. Blood tests found: C protein = 253 mg/L, neutrophils = 19 G/L. Two blood cultures were performed. The knee X-ray showed an edema. Blood cultures were positive in 10h with gram positive cocci in grape-like clusters. Knee puncture found a suppurating liquid, and direct examination was similar to blood cultures. The microbiologist performed a rapid diagnostic test that found a *S. aureus* with no methicillin resistance. This patient does not have any allergy and is in a good general shape.

14. Given the suspicion of acute arthritis on native joint, do you consider a procedure to decrease bacterial inoculum in addition to the medical treatment?
    - Yes
    - No

15. What procedure do you chose?
    - Surgery with synovectomy
    - Joint puncture(s)
    - Joint puncture, followed with surgery if progression is not favorable

16. Surgery with synovectomy
    - Arthroscopy
    - Arthrotomy

17. The following questions concern antibiotic prescription. Are you concerned with those questions?
    - Yes
    - No

A surgery with synovectomy, debridement, and microbiological samples is performed, and identifies a *S. aureus* with only a penicillin G resistance. The trans-esophageal echography does not show any argument for acute endocarditis. Blood culture after 72 hours of antibiotic treatment is sterile.

18. What antibiotic treatment do you start after surgery?

- No IV treatment, only documented oral antibiotic right after surgery
- IV treatment right after surgery

19. If you chose the IV treatment right after surgery:
    - IV treatment for 3 to 5 days right after surgery
    - IV treatment for 6 to 14 days right after surgery
    - IV treatment for 15 to 21 days right after surgery
    - Other (please specify) [open answer]
    -

20. If blood cultures would have been negative, would you have shortened the length of IV treatment?
    - Yes
    - No

21. Would you suggest using for this IV treatment an anti-staphylococcal β-lactam antibiotic (no known allergy)?
    - Yes
    - No

22. If you are suggesting an IV anti-staphylococcal β-lactam antibiotic, what is your choice:
    - Penicillin M
    - First generation cephalosporin

23. What is your suggestion as another treatment right after surgery?
    [open answer]

24. With a *S. aureus* acute arthritis on native joint, do you systematically consider a bi-antimicrobial therapy for oral treatment?
    - Yes
    - No (What is your suggestion?) [open answer]

25. In this situation, in case of a bi-antimicrobial therapy for oral treatment, would you choose rifampicin + fluoroquinolone
    - Yes
    - No, single antimicrobial therapy
    - No, other bi-antimicrobial therapy, what is your suggestion [open answer]

26. In this situation, would you consider after debridement, an oral single antimicrobial therapy?
    - No
    - Yes, with clindamycin
    - Yes, with levofloxacin or ciprofloxacin
    - Yes, with cotrimoxazole
    - Yes, with doxycycline
    - Other (please specify) [open answer]

27. What total length of antimicrobial therapy would you suggest after surgery?
    - 3 weeks
    - 4 weeks
    - 6 weeks
    - 8 weeks
    - 12 weeks
    - Other (please specify) [open answer]

28. Would you have prescribe an oral corticosteroid therapy to this patient?
    - Yes
    - No

CLINICAL CASE N°2 (Q1C2)

Twelve months later, you receive the same patient in consultation. He presents a 5° genu flexum, and a limited 75° knee bending. Clinical examination of the knee does not find any inflammatory sign, there is no joint effusion, there is no fever. C protein is 3.2 mg/L. There is an indication for knee arthroplasty (cf x-ray)

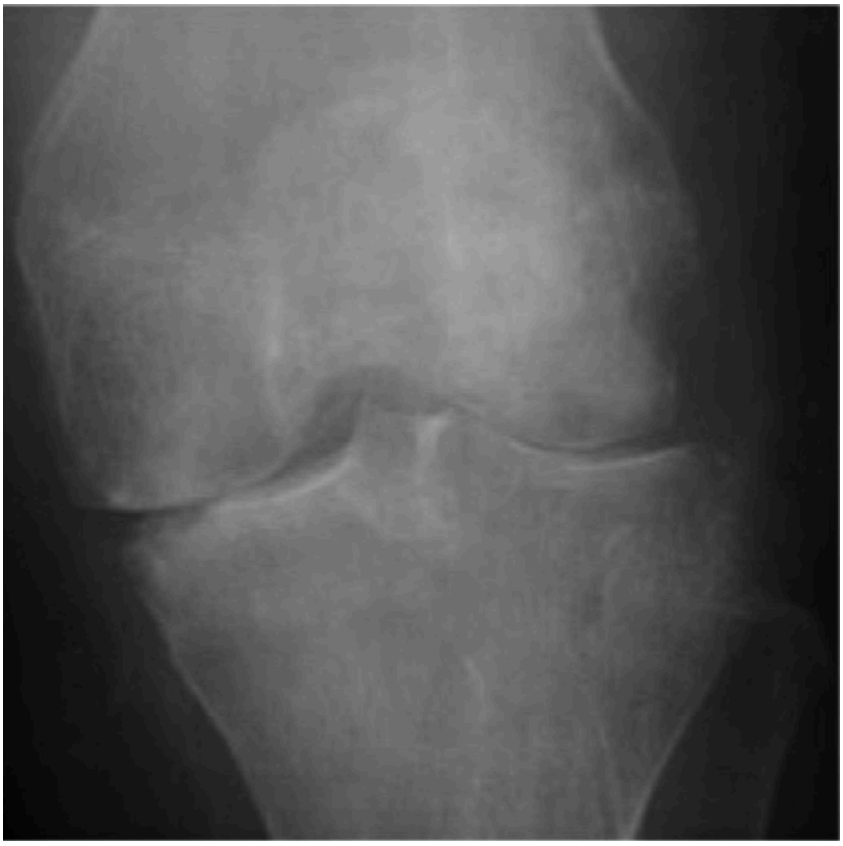

29. Do you refer this case to the multidisciplinary meeting dedicated to PJI before the arthroplasty?
    - Yes
    - No

30. Does the comorbidity of acute arthritis change the decision for the arthroplasty?
    - Yes

- No

31. Do you realize other iconographic exams for a knee arthroplasty?
    - Yes
    - No

32. What additional exams do you ask for? [several answers are possible]
    - Knee ultrasound
    - CT-scan
    - MRI
    - Bone scintigraphy
    - Indium white blood cell scan
    - LeukoScan
    - PET imaging

33. Do you realize a pre-operative knee puncture?
    - Yes
    - No

34. Before surgery, do you realize a nasal swab for the diagnosis of *S. aureus* carriage, followed with a MUPIROCIN treatment?
    - Yes
    - No

35. Do you systematically realize a MUPIROCIN decontamination, independently from a nasal swab?
    - Yes
    - No

36. What arthroplasty would you recommend in this situation?
    - One-stage arthroplasty strategy
    - Two-stage arthroplasty strategy (resection, microbiological samples, spacer, then prosthesis implantation)
    - The choice of surgery is leaded with a systematic pre-operative knee puncture for bacteriological culture
    - The choice of surgery is leaded with the results of knee imaging

37. In this situation, is the delay for prosthesis implantation independent from the results of microbiological samples?
    - Yes
    - No

38. Would you realize a sample for histologic analyze?
    - Yes
    - No

39. In this situation, at the time of arthroplasty, would you use a prophylactic antimicrobial therapy?
    - Yes
    - No

40. Would you start an antimicrobial therapy, while waiting for the results of microbiological samples?
    - Yes
    - No

41. If yes,
    - Broad-spectrum antimicrobial therapy
    - IV anti-staphylococcal antimicrobial therapy
    - Single oral anti-staphylococcal antimicrobial therapy leaded with the previous microbiological results
    - Two oral anti-staphylococcal antibiotics leaded with the previous microbiological results
