# Supplementary material for: Management of prosthetic joint infections in France: a national audit to identify key situations requiring innovation and homogenization
Source: BMC Infect Dis. 2021 May 1;21:401. doi: 10.1186/s12879-021-06075-x (PMC8088082; doi:10.1186/s12879-021-06075-x)
Supplement: Supplementary file 2 — Additional file 2. [file 12879_2021_6075_MOESM2_ESM.pdf]

Title: Management of prosthetic joint infections in France: a national audit to identify key situations requiring innovation and homogenization

Supplementary material questionnaires

Authors

Marion Le Maréchal[1-2], Zoé Cavalli[3], Cécile Batailler[4], Jean-François Gonzalez[5], André Ferreira[6], Sébastien Lustig[4], Tristan Ferry[4-7], Johan Courjon[8-9†] on the behalf of the scientific committee of CRIOAc

1. Service de Maladies Infectieuses, CHU de Grenoble
2. Université de Grenoble-Alpes
3. Service de Maladies Infectieuses, CHR de Metz-Thionville
4. CRIOAc Lyon
5. Service de chirurgie orthopédique et chirurgie du sport, Institut universitaire locomoteur et du sport (IULS), Hôpital Pasteur 2, Centre Hospitalier Universitaire de Nice, Nice, France
6. Société Française de chirurgie de la hanche et du genou
7. Université Claude Bernard Lyon 1, Lyon, France.
8. Infectiologie, Hôpital Archet 1, Centre Hospitalier Universitaire de Nice
9. Université Côte d'Azur, CHU, INSERM, C3M, France

[†]**Corresponding author:** Johan Courjon, email: courjon.j@chu-nice.fr

- Postal address: CHU de Nice, Hôpital Archet 1 Infectiologie 151 route de St Antoine de Ginestière 06200 NICE
- Phone number: +33 (0)4 92 03 54 61 Fax number +33 (0)4 92 03 90 66

Supplementary material 2 – Questionnaire 2

1. What is the name of the hospital you are working in?
   [Open answer]

2. Is your hospital a reference center in the management of prosthetic joint infection (PJI) *(Centre de Référence des Infections Ostéo-Articulaires complexes (CRIOACs))*
   - Yes
   - No

3. What is the name of your hospital?
   [Drop-down menu]

4. How many meetings dedicated to PJI are organized per month in your hospital?
   [Open answer]

5. Is there a meeting dedicated to PJI in your hospital?
   - Yes
   - No

6. How many meetings dedicated to PJI are organized per month in your hospital?
   [Open answer]

7. Do you refer to the reference center you are depending on for an opinion on complex PJI?
   - Yes
   - No

8. What is the reference center you are referring to?
   [Drop-down menu]

9. Are you answering as an individual or during a multidisciplinary meeting dedicated to PJI?
   - Individual
   - Multidisciplinary meeting dedicated to PJI

10. What is your specialty?
    - Surgeon
    - Anesthetist
    - Infectious diseases specialist
    - Internal medicine specialist
    - Physical therapy physician
    - Rheumatologist
    - Microbiologist
    - Other [Open answer]

11. If you are a surgeon, what is your specialty?

[Open answer]

12. If you are answering during a multidisciplinary meeting dedicated to PJI, what are the specialties of the attending physicians?
    [Open answer]

13. How many PJI do you manage?
    - More than 5 a week
    - 1 to 5 a week
    - 1 to 5 a month
    - Less than 1 a month

CLINICAL CASE N°1 (Q2C1)

A Z, 70 years-old man received four months ago a first right hip prosthesis (no bone cement) for a severe hip arthrosis. Right after surgery, he presented a drainage from the scar. At the time, no microbiological sample had been realized. The patient received a seven days oral treatment with CLOXACILLIN, and evolution was favorable. At your consultation, the patient presents a right hip pain, with no fever those last weeks. Cutaneous examination did not find any cellulitis nor sinus tract. You asked for a hip ultra-sound. Given the joint effusion, the radiologist realized a hip puncture. The bacteriologist informed you that there was a S. aureus with no resistance to methicillin. This patient does not have any comorbidities beside this arthroplasty. Despite the hip pain, he is totally independent in his daily activities.

1: Staphylococcus aureus :

MLSb inductible

	1: S.aureus
Pénicilline G	R
Oxacilline	S
Céfoxitine	S
Ceftaroline	S
Kanamycine	S
Gentamicine	S
Tobramycine	S
Ofloxacine	S
Lévofloxacine	S
Tétracycline	S
Tigécycline	S
Erythromycine	R
Lincomycine	S
Clindamycine	S
Pristinamycine	S
Linezolide	S
Furanes	S
Cotrimoxazole	S
Rifampicine	S
Acide fusidique	S

14. If the pre-operative puncture is positive with a non-commensal bacteria, is the choice of your antimicrobial therapy leaded with this microbiological result?

- Yes
- No

15. What strategy would you adopt?
    - One-stage exchange strategy
    - Two-stage exchange strategy
    - Arthrotomy with synovectomy for debridement, and removal of mobile components
    - Other (please specify) [open answer]

16. Does the choice of surgical strategy will change the length of antimicrobial therapy?
    - Yes
    - Non
    - Not concerned with medical treatment decisions

17. What length of antimicrobial therapy would you suggest after debridement and prosthesis retention (no other bacteria was found than the S. aureus with no meticillin resistance)?
    - 4-6 weeks
    - 6-8 weeks
    - 8-12 weeks
    - 6 months
    - Suppressive therapy

18. In this situation, with a staphylococcal prosthesis infection, would you suggest a bi-antimicrobial therapy for the oral treatment?
    - Yes
    - No

19. Would you give priority to an antimicrobial therapy that includes rifampicin?
    - Yes
    - No

20. What anti-staphylococcal antimicrobial therapy would you suggest if the patient is allergic to rifampicin? [several answers are possible]
    - FLUOROQUINOLONE
    - CLINDAMYCIN
    - COTRIMOXAZOLE
    - DOXYCYCLINE
    - FUCIDIC ACID
    - LINEZOLID
    - No prescription of RIFAMPICINE

21. What would be your first choice of antimicrobial therapy in this situation (MLSB inducible phenotype)?

- FLUOROQUINOLONE
- CLINDAMYCIN

- COTRIMOXAZOLE
- DOXYCYCLINE
- FUCIDIC ACID
- LINEZOLID
- RIFAMPICINE

22. What would be your first choice of antimicrobial therapy without an MLSB inducible phenotype?
- FLUOROQUINOLONE
- CLINDAMYCIN
- COTRIMOXAZOLE
- DOXYCYCLINE
- FUCIDIC ACID
- LINEZOLID
- RIFAMPICINE

23. In the situation of an allergy to one of this therapy, what would be your second choice (no MLSB inducible phenotype)?
- FLUOROQUINOLONE
- CLINDAMYCIN
- COTRIMOXAZOLE
- DOXYCYCLINE
- FUCIDIC ACID
- LINEZOLID
- RIFAMPICINE

24. In the situation of an allergy to one of this therapy, what would be your second choice (MLSB inducible phenotype)?
- FLUOROQUINOLONE
- CLINDAMYCIN
- COTRIMOXAZOLE
- DOXYCYCLINE
- FUCIDIC ACID
- LINEZOLID
- RIFAMPICINE

25. Does the choice of surgical strategy will change the length of antimicrobial therapy?
    - Yes
    - Non
    - Not concerned with medical treatment decisions

26. What length of antimicrobial therapy would you suggest after one-stage exchange strategy (no other bacteria was found than the S. aureus with no methicillin resistance)?
    - 4-6 weeks
    - 6-8 weeks
    - 8-12 weeks

- 6 months
- Suppressive therapy

27. In this situation, with a staphylococcal prosthesis infection, would you suggest a bi-antimicrobial therapy for the oral treatment?
    - Yes
    - No

28. Would you give priority to an antimicrobial therapy that includes RIFAMPICINE?
    - Yes
    - No

29. what anti-staphylococcal antimicrobial therapy would you suggest if the patient is allergic to rifampicin? [several answers are possible]
    - FLUOROQUINOLONE
    - CLINDAMYCIN
    - COTRIMOXAZOLE
    - DOXYCYCLINE
    - FUCIDIC ACID
    - LINEZOLID
    - No prescription of RIFAMPICINE

30. What would be your first choice of antimicrobial therapy in this situation (MLSB inducible phenotype)?

- FLUOROQUINOLONE
- CLINDAMYCIN
- COTRIMOXAZOLE
- DOXYCYCLINE
- FUCIDIC ACID
- LINEZOLID
- RIFAMPICINE

31. What would be your first choice of antimicrobial therapy without an MLSB inducible phenotype?

- FLUOROQUINOLONE
- CLINDAMYCIN
- COTRIMOXAZOLE
- DOXYCYCLINE
- FUCIDIC ACID
- LINEZOLID
- RIFAMPICINE

32. In the situation of an allergy to one of this therapy, what would be your second choice (no MLSB inducible phenotype)?

- FLUOROQUINOLONE
- CLINDAMYCIN
- COTRIMOXAZOLE

- DOXYCYCLINE
- FUCIDIC ACID
- LINEZOLID
- RIFAMPICINE

33. In the situation of an allergy to one of this therapy, what would be your second choice (MLSB inducible phenotype)?
- FLUOROQUINOLONE
- CLINDAMYCIN
- COTRIMOXAZOLE
- DOXYCYCLINE
- FUCIDIC ACID
- LINEZOLID
- RIFAMPICINE

34. In this situation, while changing the prosthesis, would you recommend using a prophylactic antimicrobial therapy before microbiological samples?
- Yes
- No

35. Does the choice of surgical strategy will change the length of antimicrobial therapy?
    - Yes
    - Non
    - Not concerned with medical treatment decisions

36. What length of antimicrobial therapy would you suggest after two-stage exchange strategy (no other bacteria was found than the S. aureus with no methicillin resistance)?
    - 4-6 weeks
    - 6-8 weeks
    - 8-12 weeks
    - 6 months
    - Suppressive therapy

37. What strategy do you suggest for the reimplantation of the prosthesis?
    - During antimicrobial therapy
    - After 10-15 days without any antimicrobial therapy
    - Other (please specify [open answer]

38. In this situation, with a staphylococcal PJI, with a prosthesis, would you suggest a bi-antimicrobial therapy for the oral treatment?
    - Yes
    - No

39. Would you give priority to an antimicrobial therapy that includes RIFAMPICINE?
    - Yes
    - No

40. What anti-staphylococcal antimicrobial therapy would you suggest if the patient is allergic to rifampicin? [several answers are possible]
    - FLUOROQUINOLONE
    - CLINDAMYCIN
    - COTRIMOXAZOLE
    - DOXYCYCLINE
    - FUCIDIC ACID
    - LINEZOLID
    - No prescription of RIFAMPICINE

41. What would be your first choice of antimicrobial therapy in this situation (MLSB inducible phenotype)?

- FLUOROQUINOLONE
- CLINDAMYCIN
- COTRIMOXAZOLE
- DOXYCYCLINE
- FUCIDIC ACID
- LINEZOLID
- RIFAMPICINE

42. What would be your first choice of antimicrobial therapy without an MLSB inducible phenotype?

- FLUOROQUINOLONE
- CLINDAMYCIN
- COTRIMOXAZOLE
- DOXYCYCLINE
- FUCIDIC ACID
- LINEZOLID
- RIFAMPICINE

43. In the situation of an allergy to one of this therapy, what would be your second choice (no MLSB inducible phenotype)?

- FLUOROQUINOLONE
- CLINDAMYCIN
- COTRIMOXAZOLE
- DOXYCYCLINE
- FUCIDIC ACID
- LINEZOLID
- RIFAMPICINE

44. In the situation of an allergy to one of this therapy, what would be your second choice (MLSB inducible phenotype)?

- FLUOROQUINOLONE
- CLINDAMYCIN
- COTRIMOXAZOLE
- DOXYCYCLINE

- FUCIDIC ACID
- LINEZOLID
- RIFAMPICINE

45. If you chose to implant the new prosthesis at the end of antimicrobial therapy, do you start a new antimicrobial therapy after surgery, while waiting for the results of microbiological samples?
- Yes
- No

46. What new antimicrobial therapy do you start? [several answers possible]
- Anti-staphylococcal antimicrobial therapy
- Broad-spectrum antimicrobial therapy
- Antimicrobial therapy leaded with a pre-operative puncture

47. For this arthroplasty, after second stage, would you prescribe a prophylactic antimicrobial therapy before microbiological samples?
- Yes (what antimicrobial therapy would you choose) [open answer]
- No

CLINICAL CASE N°2 (Q2C2)

Mr Z, 69 years-old, 96kg, received three years ago a left cemented knee prosthesis for arthrosis. One year after surgery, he started to feel pain on this same knee. You see him nine months after pain onset, and you observe pain with and without movement. There is a knee effusion, without any inflammatory signs nor sinus tract.

At CT-scan, the implant looks moderately oversized and some effusion is detected. Microbiological exam of the knee effusion after puncture found gram positive cocci in chains, identified as *Enterococcus faecalis*, sensitive to amoxicillin. X-ray does not find any loosening of the prosthesis.

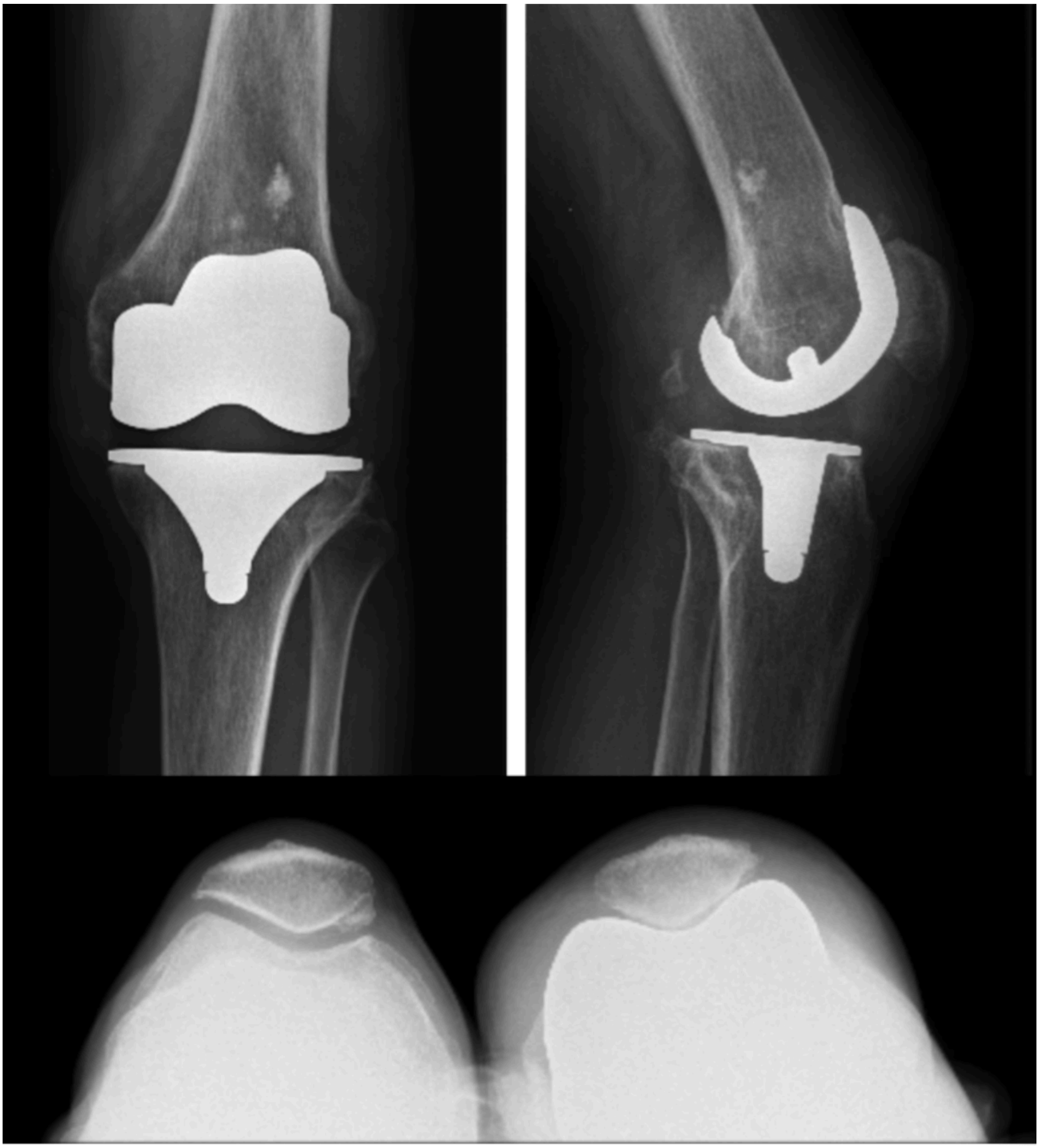

48. What surgical management would you chose? [single answer]
- Arthrotomy with synovectomy for debridement, and removal of mobile components
- One-stage exchange strategy with a non-cemented prosthesis
- One-stage exchange strategy with an antibiotic loaded bone cement (industrial gentamicin + vancomycin or gentamicin + clindamycin)
- One-stage exchange strategy with an antibiotic loaded bone cement (industrial gentamicin
- Two-stage exchange strategy without using a spacer
- Two-stage exchange strategy using a non-antibiotic loaded spacer
- Two-stage exchange strategy using an antibiotic loaded spacer (gentamicin)
- Two-stage exchange strategy using an antibiotic loaded spacer (industrial gentamicin + manual vancomycin)
