# Supplementary material for: Management of prosthetic joint infections in France: a national audit to identify key situations requiring innovation and homogenization
Source: BMC Infect Dis. 2021 May 1;21:401. doi: 10.1186/s12879-021-06075-x (PMC8088082; doi:10.1186/s12879-021-06075-x)
Supplement: Supplementary file 3 — Additional file 3. [file 12879_2021_6075_MOESM3_ESM.pdf]

Title: Management of prosthetic joint infections in France: a national audit to identify key situations requiring innovation and homogenization

Supplementary material questionnaires

Authors

Marion Le Maréchal[1-2], Zoé Cavalli[3], Cécile Batailler[4], Jean-François Gonzalez[5], André Ferreira[6], Sébastien Lustig[4], Tristan Ferry[4-7], Johan Courjon[8-9†] on the behalf of the scientific committee of CRIOAc

1. Service de Maladies Infectieuses, CHU de Grenoble
2. Université de Grenoble-Alpes
3. Service de Maladies Infectieuses, CHR de Metz-Thionville
4. CRIOAc Lyon
5. Service de chirurgie orthopédique et chirurgie du sport, Institut universitaire locomoteur et du sport (IULS), Hôpital Pasteur 2, Centre Hospitalier Universitaire de Nice, Nice, France
6. Société Française de chirurgie de la hanche et du genou
7. Université Claude Bernard Lyon 1, Lyon, France.
8. Infectiologie, Hôpital Archet 1, Centre Hospitalier Universitaire de Nice
9. Université Côte d'Azur, CHU, INSERM, C3M, France

[†]**Corresponding author:** Johan Courjon, email: courjon.j@chu-nice.fr

- Postal address: CHU de Nice, Hôpital Archet 1 Infectiologie 151 route de St Antoine de Ginestière 06200 NICE

- Phone number: +33 (0)4 92 03 54 61 Fax number +33 (0)4 92 03 90 66

Supplementary material 3 – Questionnaire 3

1. What is the name of the hospital you are working in?
   [Open answer]

2. Is your hospital a reference center in the management of prosthetic joint infection (PJI) *(Centre de Référence des Infections Ostéo-Articulaires complexes (CRIOACs))*
   - Yes
   - No

3. What is the name of your hospital?
   [Drop-down menu]

4. How many meetings dedicated to PJI are organized per month in your hospital?
   [Open answer]

5. Is there a meeting dedicated to PJI in your hospital?
   - Yes
   - No

6. How many meetings dedicated to PJI are organized per month in your hospital?
   [Open answer]

7. Do you refer to the reference center you are depending on for an opinion on complex PJI?
   - Yes
   - No

8. What is the reference center you are referring to?
   [Drop-down menu]

9. Are you answering as an individual or during a multidisciplinary meeting dedicated to PJI?
   - Individual
   - Multidisciplinary meeting dedicated to PJI

10. What is your specialty?
    - Surgeon
    - Anesthetist
    - Infectious diseases specialist
    - Internal medicine specialist

- Physical therapy physician
- Rheumatologist
- Microbiologist
- Other [Open answer]

11. If you are a surgeon, what is your specialty?
    [Open answer]

12. If you are answering during a multidisciplinary meeting dedicated to PJI, what are the specialties of the attending physicians?
    [Open answer]

13. How many PJI do you manage?
    - More than 5 a week
    - 1 to 5 a week
    - 1 to 5 a month
    - Less than 1 a month

14. Have you heard about the following recommendations on the management of PJI?
    - French National Infectious Diseases Society recommendations 2009
    - Infectious Diseases Society of America recommendations 2013
    - French Health authorities' recommendations 2014
    - Spanish recommendations SEIMC of 2017
    - International Philadelphia recommendations 2018

15. Have you read the following recommendations on the management of PJI?
    - French National Infectious Diseases Society recommendations 2009
    - Infectious Diseases Society of America recommendations 2013
    - French Health authorities' recommendations 2014
    - Spanish recommendations SEIMC of 2017
    - International Philadelphia recommendations 2018

CLINICAL CASE N°1 (Q3C1)

Mr X, 70 years-old, presents diabetes mellitus and a comorbidity of a *Streptococcus* spp. infection of his left knee prosthesis five years ago. At this time, he benefited from a prosthesis 2-stage exchange strategy. He recently underwent a prosthesis exchange for aseptic loosening (per-operative microbiological samples were negative). You see him 21 days after surgery for a wound drainage: he has a 38.1°C body temperature, a knee effusion, the drainage is suppurating. Your first suspicion is a prosthesis infection.

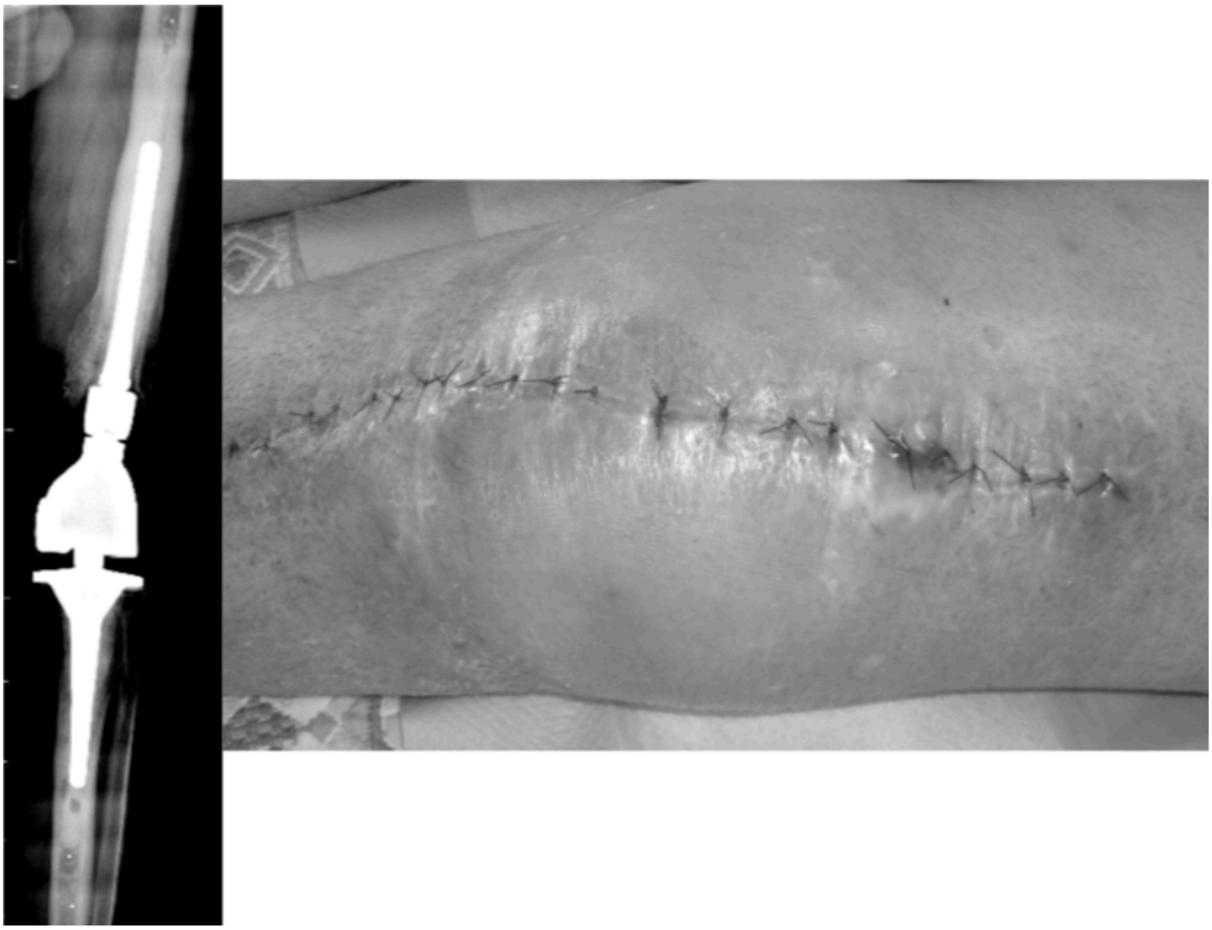

16. Do you perform a knee puncture?
    - Yes
    - No

17. If you perform a knee puncture, what analyses are asking?
    - Synovial fluid inoculation in blood culture
    - Synovial fluid inoculation in sample container
    - Synovial fluid inoculation for cytochemistry analysis
    - Synovial fluid inoculation for cytologic analysis

18. What is time limit for patient care ?
    - Within 24h
    - Within 72h
    - Within the week
    - Within the month

19. What surgical strategy is usually considered for acute post-operative prosthesis infection?
    - Debridement via an arthroscopy
    - Debridement with removal of mobile components via an open arthrotomy

- One-stage exchange strategy without cement
- Prosthesis and cement removal as the first stage of a two-stage exchange strategy

20. What strategy are you going to chose ?
    - Debridement via an arthroscopy
    - Debridement with removal of mobile components via an open arthrotomy
    - Prosthesis and cement removal for a one-stage exchange strategy
    - Prosthesis and cement removal as the first stage of a two-stage exchange strategy

21. Following questions concern antibiotic prescription in the case of a PJI, if you are not concerned, chose "next questionnaire"
    - Continue the questionnaire
    - Next questionnaire

22. The surgeon performed a debridement via an open arthrotomy, but could not change the polyethylene inserts. What post-operative empirical antimicrobial therapy would you choose?
    - Anti-Gram negative:
        - Carbapenem
        - Cefepime
        - Ceftazidime
        - Piperacillin/tazobactam
    - Anti-Gram positive:
        - Daptomycin
        - Linezolid
        - Vancomycin
        - Ceftobiprole
        - Gentamicin
    - Other [Open answer]

23. Is the choice for post-operative empirical antimicrobial therapy written in a local antibiotic prescription guide?
    - Yes
    - No

24. The patient received a cefepime and vancomycin antibiotic treatment. All microbiological samples finally isolated a *Staphylococcus aureus*. Blood cultures are negative. What is your decision 48h after surgery (antimicrobial susceptibility testing is not available yet)?
    - Sustain of the same empirical antibiotic therapy

- Sustain of the same empirical antibiotic therapy with rifampicin in addition
- Switch for penicillin M or first-generation cephalosporin
- Switch of the anti-Gram negative beta-lactam antibiotic for penicillin M or first generation cephalosporin and sustain of the anti-gram positive empirical antimicrobial therapy effective against MRSA.
- Switch for penicillin M or first-generation cephalosporin with rifampicin in addition
- Sustain only of the anti-Gram positive empirical antimicrobial therapy active against MRSA
- Other [please specify]

25. The next day, you receive the antimicrobial susceptibility testing. The Staphylococcus aureus is susceptible to meticilline, and all other antibiotics, except penicillin G. There are no other bacteria identified five days after surgery. When would you switch for an exclusive oral antimicrobial therapy?
    - Immediate switch
    - Five days after surgery
    - Between five and eight days after surgery
    - Between eight and twelve days after surgery
    - Between twelve and fifteen days after surgery

26. If you want to sustain, at least partly, IV antimicrobial treatment at day five after surgery, what would be your choice?
    - Penicillin M or first-generation cephalosporin alone
    - Penicillin M or first-generation cephalosporin and oral fluoroquinolone
    - Penicillin M or first-generation cephalosporin and oral rifampicin
    - Penicillin M or first-generation cephalosporin and oral clindamycin
    - Penicillin M or first-generation cephalosporin and another antibiotic
    - Other [please specify]

27. Generally, for PJI, can you specify your preference for fluoroquinolones depending on the bacteria:
    - Staphylococcus
        - Levofloxacin
        - Ofloxacin
    - Streptococcus
        - Levofloxacin
        - Ofloxacin
    - Enterobacteria (sensitive to nalidixic acid)
        - Ciprofloxacin
        - Levofloxacin
        - Ofloxacin

- Enterobacteria (resistant to nalidixic acid)
    - Ciprofloxacin as a single therapy
    - Ciprofloxacin as a bi-antimicrobial therapy
    - Levofloxacin as a bi-antimicrobial therapy
    - Ofloxacin as a bi-antimicrobial therapy
    - No fluoroquinolones
- *Pseudomonas aeruginosa*
    - Ciprofloxacin
    - Ofloxacin

28. The patient received seven days of IV penicillin M + levofloxacin. He does not have any fever and the surgical wound improves appropriately. What is your choice for oral antimicrobial therapy?
    - Fluoroquinolone – clindamycin
    - Fluoroquinolone – rifampicin
    - Clindamycin – rifampicin
    - Cotrimoxazole – rifampicin
    - Fucidic acid – rifampicin
    - Other [please specify]

29. The patient is treated with a bi-antimicrobial therapy levofloxacin – rifampicin? (weight 85kg)
    - 300mg twice a day
    - 450mg twice a day
    - 600mg once a day
    - 600mg twice a day
    - 900mg once a day
    - 900mg twice a day

30. What dosage do you use for levofloxacin? (no kidney failure, weight 85kg)
    - 500mg once a day
    - 500mg twice a day
    - 750mg once a day
    - 750mg twice a day

31. What duration of antibiotic treatment do you choose?
    - Six weeks
    - Three months
    - Six months
    - Suppressive antibiotic treatment following initial treatment

CLINICAL CASE N°2

Ms. Y, 65 years-old, 60kg, benefited from a left hip prosthesis without cement nine months ago. Since, she reports persistent pain when she walks. Scar examination is normal, there is no cellulitis, nor fever. Indium white blood cell scan has arguments for an infection. CT-scan finds a hip swelling. Direct examination of the synovial puncture finds Gram-positive cocci in chains. X-ray finds a well-fixed hip prosthesis.

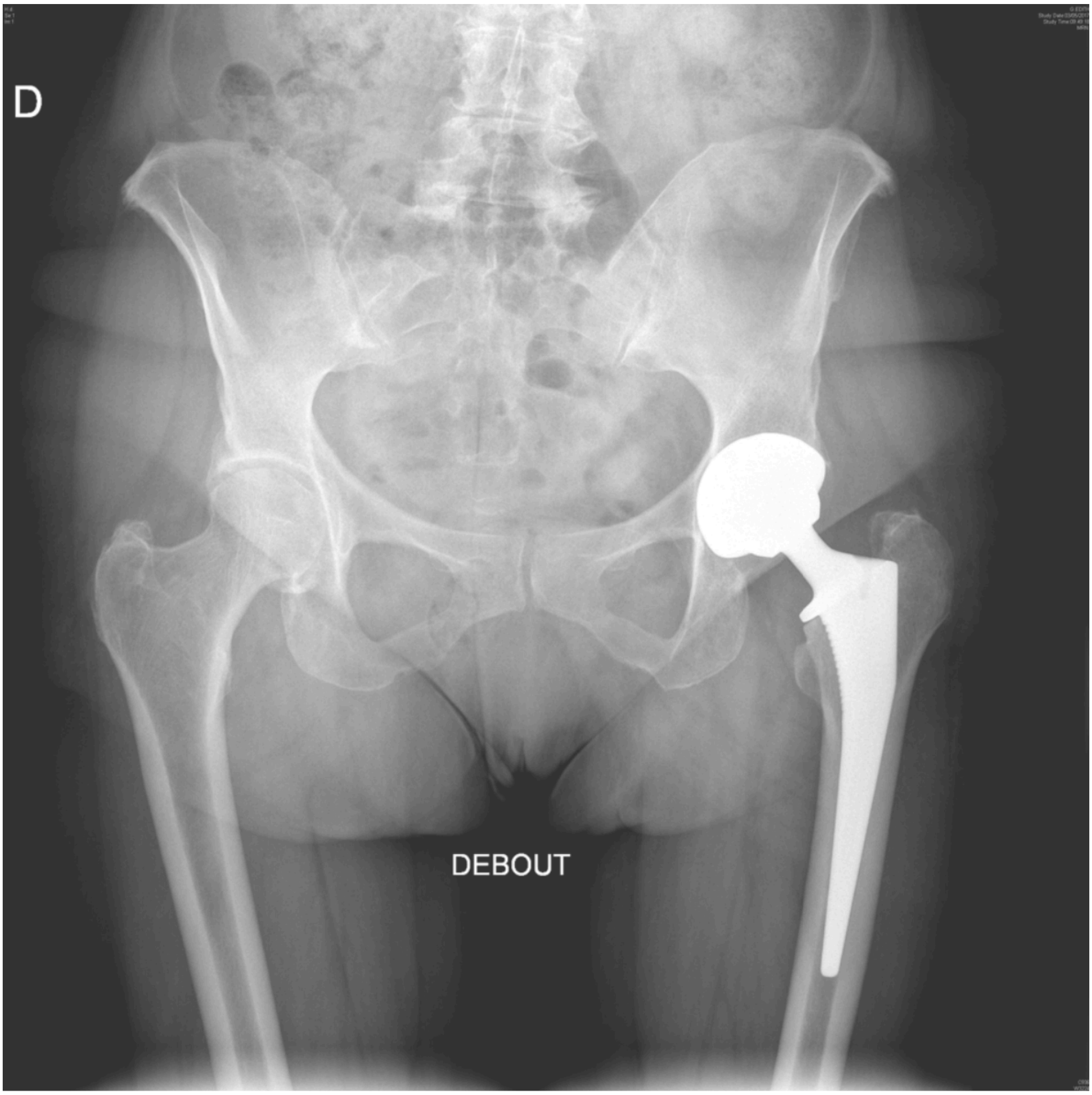

32. What surgical strategy do you suggest?
    - Debridement with removal of mobile components via an open arthrotomy
    - One-stage exchange strategy with a non-cemented prosthesis
    - One-stage exchange strategy with an antibiotic loaded cemented prosthesis (gentamicin)
    - One-stage exchange strategy with an antibiotic loaded cemented prosthesis (gentamicin+clindamycin or gentamicin+vancomycin)

- One-stage exchange strategy with an antibiotic loaded cemented prosthesis (gentamicin + vancomycin)
- Two-stage exchange strategy without a spacer
- Two-stage exchange strategy with a spacer without antibiotics
- Two-stage exchange strategy with an antibiotic loaded cemented spacer (gentamicin)
- Two-stage exchange strategy with an antibiotic loaded cemented spacer (gentamicin+clindamycin or a gentamicin+vancomycin)
- Two-stage exchange strategy with an antibiotic loaded cemented spacer (gentamicin+vancomycin)

33. Culture of synovial puncture finds a *Streptococcus agalactiae* sensitive to amoxicillin, erythromycin, clindamycin and levofloxacin. Do you prescribe an IV amoxicillin treatment before surgery?
    - Yes
    - No

34. The patient receives a one-stage prosthesis exchange strategy. What immediate post-operative antibiotic do you prescribe?
    - IV amoxicillin (single therapy) with a minimum of 100mg/kg dosage
    - IV amoxicillin (minimum of 100mg/kg dosage) + vancomycin
    - IV amoxicillin (minimum of 100mg/kg dosage) + daptomycin
    - IV amoxicillin (minimum of 100mg/kg dosage) + linezolid
    - Amoxicillin + another antibiotic efficient against streptococcus (rifampicin, levofloxacin, clindamycin, or pristinamycin)
    - Vancomycin (single therapy)
    - Daptomycin (single therapy)
    - Linezolid (single therapy)
    - Piperacillin/tazobactam + vancomycin
    - Piperacillin/tazobactam + daptomycin
    - Piperacillin/tazobactam + linezolid
    - Other [Please specify]

35. Five days after surgery, the patient is treated with IV amoxicillin, the surgical wound is clean. Per-operative identified bacteria is identical to the Streptococcus agalactiae in the pre-operative synovial puncture. When do you switch antibiotics for an exclusive oral treatment?
    - Five days after surgery
    - Between five and eight days after surgery
    - Between eight and twelve days after surgery
    - Between twelve and fifteen days after surgery

36. What antibiotic do you choose for oral switch?
   - Amoxicillin
   - Amoxicillin + rifampicin
   - Amoxicillin + fluoroquinolone
   - Amoxicillin + clindamycin
   - Clindamycin
   - Clindamycin + rifampicin
   - Fluoroquinolone
   - Fluoroquinolone + rifampicin
   - Fluoroquinolone + clindamycin
   - Fluoroquinolone
   - Other [please specify]

37. How long do you prescribe the antibiotics for?
   - Six weeks
   - Eight weeks
   - Twelve weeks

38. What portal of entry do you consider?
   - ORL
   - Urinary
   - Digestive
   - Cutaneous
   - Pulmonary
   - None
   - Other [Please specify]
